# Improved detection of *BRAF* V600E using allele-specific PCR coupled with external and internal controllers

**DOI:** 10.1038/s41598-017-14140-2

**Published:** 2017-10-23

**Authors:** Zhao Yang, Na Zhao, Dong Chen, Kun Wei, Ning Su, Jun-Fu Huang, Han-Qing Xu, Guang-Jie Duan, Wei-Ling Fu, Qing Huang

**Affiliations:** 1 Department of Laboratory Medicine, Southwest Hospital, Third Military Medical University, Chongqing, 400038 P. R. China; 2 Department of Laboratory Medicine; 302 hospital of PLA, Chongqing, 100039 P. R. China; 3 Institute of Pathology and Southwest Cancer Center, Southwest Hospital, Third Military Medical University, Chongqing, 400038 P. R. China

## Abstract

Although traditional allele-specific PCR (tAS-PCR) is a common screening method for *BRAF* V600E mutations, its lower amplification specificity and mutation selectivity have limited its clinical applications. We hypothesize that these limitations are associated with the weaker specificities of allele-specific primers and the thermodynamic driving forces of DNA polymerase. We used three strategies to circumvent these limitations, namely, modifying allele-specific primers, introducing a competitive external allele-specific controller (i.e., cAS-PCR), and introducing a referenced internal positive controller in the cAS-PCR (i.e., rcAS-PCR). The amplification sensitivities and specificities were influenced by the position of the artificially introduced mismatched nucleotide in the allele-specific primers. Moreover, both cAS-PCR and rcAS-PCR could detect single-copy *BRAF* V600E alleles with higher mutation selectivity (0.1%) than tAS-PCR. In addition, cAS-PCR eliminated false-negative results caused by various PCR inhibitors that might be present in the DNA solutions. The rcAS-PCR could also be employed to avoid the false-negative results caused by low-abundance input templates in cAS-PCR. In conclusion, rcAS-PCR provides a rapid, simple, and low-cost method for detecting low levels of the mutated *BRAF* V600E gene.

## Introduction

The most recent cancer statistics in China shows ~4.3 million newly diagnosed invasive cancer cases in 2015, corresponding to almost 12,000 new cancer diagnoses each day^1^. Among the various cancers in China, colorectal carcinoma (CRC) is one of the five most commonly diagnosed cancers affecting both men and women^1^. Although two monoclonal antibodies targeting the epidermal growth factor receptor (EGFR), i.e., Cetuximab (Erbitux^®^, ImClone Systems) and Panitumumab (Vectibix^®^, Amgen), have been used clinically for the targeted therapy of human metastatic CRC (mCRC), these drugs only have favorable response and disease stabilization rates for ~10% and ~30% of mCRC patients, respectively^2,3^. Therefore, a cost-effective and reliable method that can accurately predict a patient’s response to these therapies is warranted.

The RAS-RAF-MAPK pathway is a major signaling pathway that triggers cell proliferation upon EGFR ligand binding by activating the *KRAS* and *BRAF* genes. Both *KRAS* and *BRAF* are required to be wild-type (WT) for the responsiveness to Panitumumab or Cetuximab therapy in mCRC^4^. In CRC patients with WT *KRAS*, ~15% have *BRAF* V600E mutations^4–7^; therefore, *BRAF* V600E mutations could account for an additional 15% of patients who are *KRAS* WT but are non-responsive to anti-EGFR monoclonal antibodies^4–7^.

Selectivity, which refers to the abilities to detect mutant (MT) alleles selectively among an excess of WT-alleles, is one of the most important methodological parameters in mutation analysis^8–10^. Selectivity is defined as the ratio of copy number between minimum detectable MT-alleles and the total inputted alleles, including both WT- and MT-alleles^8–10^. Among various methods targeting *BRAF* V600E, allele-specific PCR (AS-PCR) is the most common but always has a limited selectivity of 1–5%, i.e., it can only detect around 1–5% of MT-alleles except for one publication reported higher selectivity up to 0.3% using TaqMan-based AS-PCR system^11–13^. In traditional AS-PCR (tAS-PCR), an allele-specific (AS) nucleotide is always present at the last position of the 3′-end of the AS-primer (ASP). However, its allelic determination is often hampered by cross-hybridization between the defined genotypic ASP and the opposite templates. Although artificial mismatched nucleotides can be introduced at the *penultimate* (second to the terminal) or the *antepenultimate* (third to the terminal) position at the 3′-end of the ASP to enhance their priming specificities, these cannot always accurately discriminate between different alleles, thereby leading to false-positive results^14,15^.

In the presence of a single pair of primers in the PCR reaction mixture, the amplification of the template DNA is controlled by the thermodynamic driving force of the thermophilic DNA polymerase, thereby producing positive amplicons, often without sequence complementarity between primers and templates, resulting in non-specific amplifications between templates and mismatched primers^14,16^. In tAS-PCR, when only one genotypic ASP (e.g., MT-genotype) is included in the reaction and under the thermodynamic driving force of DNA polymerase, the single-base terminal mismatch between the primers and template can easily trigger the non-specific amplification of an input DNA having the opposite genotype (e.g., WT-genotype)^14,16^. Moreover, the weak destabilization effects of terminal mismatches can further promote non-specific amplification^15^. Although stringent reaction conditions can be used to significantly reduce or eliminate non-specific amplification, optimization is time-consuming and often unsuccessful.

In the present study, a fragment termed competitive external allele-specific controller (CEAC), which shares the same binding sequences of tAS-PCR primers targeting the human *BRAF* V600E MT-alleles, was cloned and used in the preparation of CEAC plasmids. To satisfy the requirement for the thermodynamic driving force of DNA polymerase, tAS-PCR using a CEAC plasmid (cAS-PCR) was developed to eliminate non-specific amplification that frequently occurs in the tAS-PCR system. To further monitor the input amount of sample genomic DNA (gDNA), a referenced internal positive controller (RIPC) was introduced into the reaction mixture to develop cAS-PCR with RIPC (rcAS-PCR). The results showed that non-specific amplification could be eliminated by the introduction of CEAC into the cAS-PCR system. Moreover, it was easy to monitor the amount of initial input gDNA to avoid false-negative results because of the further introduction of RIPC amplicons in the rcAS-PCR system. Compared to tAS-PCR, both cAS-PCR and rcAS-PCR showed higher specificities, selectivities, and sensitivities. Therefore, these two novel systems may be utilized in the clinical screening for oncogenic mutations.

## Materials and Methods

### Extraction of sample gDNA

The gDNA was extracted from two cell lines, i.e., A375 and SW480 (ATCC, Manassas, VA, USA), that harbor homozygous MT- and WT-alleles of the human *BRAF* V600E gene, respectively^11,17^, using a QIAamp^®^ DNA Blood Mini Kit (Qiagen, Hilden, Germany) following the manufacturer’s introductions. Hereafter, gDNA extracted from the SW480 and A375 cells are referred to as WT- and MT-gDNA in the following sections, respectively.

Formalin-fixed paraffin-embedded (FFPE) tissue blocks from Chinese mCRC patients were obtained from the Southwest Hospital (Chongqing, China). The ethics committee of Southwest Hospital approved this study including any relevant details, and written informed consent was obtained from the patients or their family members prior to sample collection. All experiments were performed in accordance with relevant guidelines and regulations. Genomic DNA was extracted from FFPE sections as described elsewhere^17^.

### Quantitative determination of the extracted gDNA

The concentration of the gDNAs extracted from cell lines or FFPE sections was quantitatively determined using a *leptin* quantitative PCR (qPCR) system. The reaction mixture contained 1× Platinum^®^ Quantitative PCR SuperMix-UDG (Invitrogen, Waltham, MA, USA), 200 nM of the forward and reverse locus-specific primers (LSP; HQ-329 and -330; Table 1), and 100 nM of locus-specific TaqMan probes (LST; 5′-(6-FAM)CGGTTTGGACTTCATTCCTGGGCTCC(BHQ1)-3′). Serial concentrations of human gDNA (Cat No. G304A; Promega, Madison, WI, USA) were used to prepare a standard curve of *leptin*. Reactions were performed on a CFX96 Real-Time PCR Detection System (Bio-Rad, Hercules, CA, USA) under the following cycling conditions: incubation at 50 °C for 2 min, denaturation at 95 °C for 30 sec, and 50 cycles of 95 °C for 15 sec and 60 °C for 1 min (with single fluorescence acquisition). The quantification cycle (*C*_q_) values were determined automatically by using CFX Manager™ Software v3.1 (Bio-Rad).

**Table 1:** Oligonucleotides used in the current tAS-PCR, cAS-PCR, and rcAS-PCR.

Targets	ID	Description	5′–3′ Sequences
Porcine	HQ-648	LST	(CY5)ACTAGCCCCATTATCAGTACTATGCCA(BHQ2)
*BRAF*	HQ-675	Forward LSP	ACTACACCTCAGATATATTTCTTCATG
	HQ-668	Reverse ASP	CCCACTCCATCGAGATTTCT
	HQ-666	Reverse ASP	CCCACTCCATCGAGATTTtT
	HQ-663	Reverse ASP	CCCACTCCATCGAGATTgCT
	HQ-467	LST	(6-FAM)ATCACCTATTTTTACTGTGAGGTCTT(BHQ1)
*Leptin*	HQ-329	Forward LSP	CAGTCTCCTCCAAACAGAAAGTCA
	HQ-330	Reverse LSP	GTCCATCTTGGATAAGGTCAGGA
	HQ-1294	LST	(Texas Red)CGGTTTGGACTTCATTCCTGGGCTCC(BHQ2)

The sizes were 147-, 89-, and 80-bp for the amplicons of CEAC, *BRAF* V600E and *leptin*, respectively. The lower-case letters in HQ-666 and -663 indicate the artificially introduced mismatched bases.

### Preparation of CEAC plasmids

Using mitochondrial DNA from *Sus scrofa* (pig, porcine) as the initial template^18^, the final amplicons of two rounds of PCR were sent to Sangon Biotech Co., Ltd. (Shanghai, China) and used in the preparation of CEAC plasmids. The amplicons of the first round using forward (5′-CCAAGGCATTTCACTACAAG-3′) and reverse (5′-GTTTGGGTTGATTGATTGTG-3′) primers were used as the templates for the second round using forward (5′-TGAGATCTACTGTTTTCCTTTACTTACTACACCTCAGATATATTTCTTCATGCCAAGGCATTTCACTACAAG-3′) and reverse (5′-AACTGTTCAAACTGATGGGACCCACTCCATCGAGATTTCTGTTTGGGTTGATTGATTGTG-3′) primers in which the underlined sequences were the same as those of the *BRAF* V600E MT-alleles. Both PCR reactions were performed in a reaction mixture containing 1 × *Ex Taq* Buffer (Mg^2+^ plus; TaKaRa, Dianian, China), 250 μM of each dNTP (TaKaRa), 0.5 U *Ex Taq* ^®^ HS DNA Polymerase (TaKaRa), and 200 nM of each primer pair. Reactions were performed on a Veriti™ 96-well Thermal Cyclers (Applied Biosystems, Forster City, CA, USA) under the following cycling conditions: denaturation at 94 °C for 5 min; followed by 35 cycles of 94 °C for 30 sec, 60 °C for 30 sec, and 72 °C for 30 sec; and a final extension step at 72 °C for 5 min.

### Screening ASP oligonucleotides targeting BRAF V600E MT-alleles in tAS-PCR

Three types of ASP oligonucleotides (i.e., HQ-668, -666, and -663; Table 1) targeting human *BRAF* V600E MT-alleles were designed and the one showing superior amplification efficiency and specificity was selected and utilized in the subsequent analyses. The reaction mixture contained 1× Platinum^®^ Quantitative PCR SuperMix-UDG (Invitrogen), 200 nM of forward LSP (HQ-675 in Table 1), 200 nM of each reverse ASP (i.e., HQ-668, -666, or -663; Table 1), 100 nM of LST (HQ-467; Table 1), and differing concentrations of WT- or MT-gDNA. Except for the increase in the number of cycles to 55 and the melting temperature (Tm) ranging from 60 °C to 67 °C, the thermal cycling conditions and data analysis methods were the same as those used for the *leptin* qPCR system.

### Optimization of tAS-PCR, cAS-PCR, and rcAS-PCR

**Figure 1:**
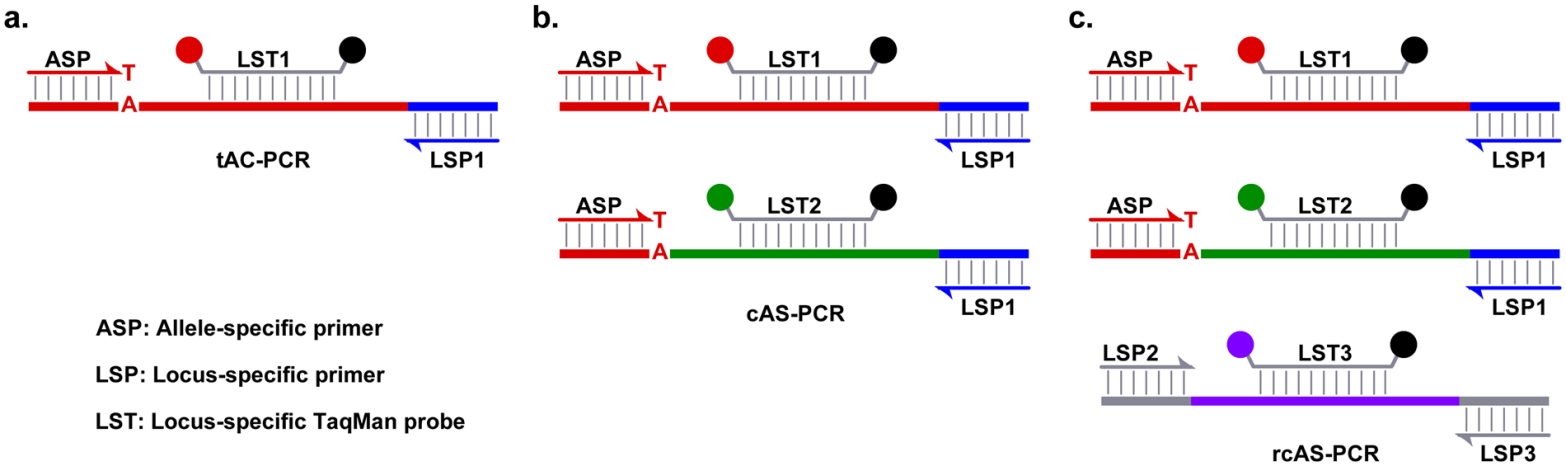
Principles of tAS-PCR, cAS-PCR, and rcAS-PCR. The principles of tAS-PCR, cAS-PCR, and rcAS-PCR are shown in panels a, b, and c, respectively. In the tAS-PCR system, only primers and probes targeting MT-alleles (e.g., V600E) were used (i.e., ASP, LSP1, and LST1). In the cAS-PCR, in addition to the oligonucleotides in the tAS-PCR system, artificially introduced external templates (i.e., CEAC plasmids) and corresponding probes (i.e., LST2) were used. In the rcAS-PCR, in addition to the oligonucleotides and artificial templates used in cAS-PCR system, additional primers and probes targeting RIPC (e.g., the *leptin* gene) were used (i.e., LSP2, LSP3, and LST3). In the current study, the corresponding oligonucleotides used were: ASP is HQ-663, -666, or -668; LSP1 is HQ-675; LSP2 is HQ-329; LSP3 is HQ-330; LST1 is HQ-467; LST2 is HQ-648; LST3 is HQ-1294 (Table 1).

Three types of real-time AS-PCR (Fig. 1) were performed: tAS-PCR, cAS-PCR (i.e., tAS-PCR with CEAC), and rcAS-PCR (i.e., cAS-PCR with RIPC). For tAS-PCR (Fig. 1a), the reaction mixture contained only LST (i.e., HQ-467; Table 1), a forward LSP (i.e., HQ-675; Table 1), and one of the reverse ASPs targeting the V600E MT-alleles (i.e., HQ-663, -666, or -668; Table 1). The cAS-PCR reactions (Fig. 1b) were conducted using the same reagent mixture as that of the tAS-PCR reaction, but additionally including the CEAC plasmids and the corresponding LST (i.e., HQ-301; Table 1). The rcAS-PCR (Fig. 1c) reactions included the same reagent mixture as that of the cAS-PCR, as well as additional LSP and LST oligonucleotides (i.e., HQ-329, -330, and -1294; Table 1) that target the RIPC (i.e., human *leptin* genes). All reaction mixtures contained 1× Platinum^®^ Quantitative PCR SuperMix-UDG (Invitrogen), the specified oligonucleotides, and templates at certain concentrations. The thermal cycling conditions and data analysis methods were the same as those of the *leptin* qPCR system.

### The Taguchi method

The concentrations of the primer pairs and probes targeting V600E, CEAC, and RIPC in the rcAS-PCR system were optimized using the Taguchi method (Tables S1 and S2)^14,19,20^. After the optimal concentrations of each factor were determined, additional experiments were performed using these conditions to confirm the results^14,19,20^. A detailed description of all calculations and equations used in the Taguchi method could be found in the Supplementary data. Each Taguchi method experiment and the corresponding confirmatory tests were performed twice, each in duplicate (Tables S1 to S3).

### Analysis of clinical samples using cAS-PCR and rcAS-PCR

The optimized cAS-PCR and rcAS-PCR conditions were used to analyze 50 clinical mCRC FFPE samples. All samples were co-analyzed with commercial available reagents, following the manufacturer’s introductions, i.e., the *BRAF* V600E Mutation Detection Kit (Amoy Diagnostics, Xiamen, China).

## Results

### Screening various ASPs targeting the BRAF V600E MT-alleles

The tAS-PCR (Fig. 1a) was performed to evaluate the specificity and amplification efficiency of the three types of ASPs targeting the V600E MT-alleles: (a) those having full complementary sequences (i.e., HQ-668), and (b) those having an artificially introduced mismatched nucleotide at the *penultimate* (i.e., HQ-666), or (c) at the *antepenultimate* positions (i.e., HQ-663) to enhance its priming specificities^15,21–23^.

**Figure 2:**
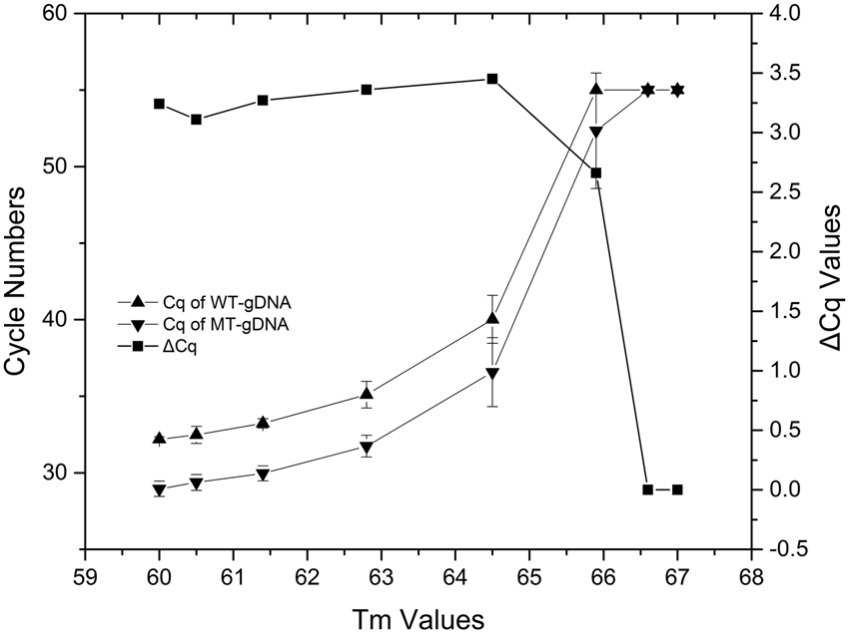
Screening ASP using tAS-PCR with gradient Tm. The *C*_q_ values (i.e., left Y-axis) of WT- or MT-gDNA at a final concentration of 50 ng and their corresponding Δ*C*_q_ values (i.e., right Y-axis) in tAS-PCR system using ASP oligonucleotide of HQ-663 under a gradient Tm ranging from 60 °C to 67 °C, as indicated on the X-axis. The results of HQ-666 and -668 show similar patterns to that of abovementioned HQ-663 (data not shown). The standard deviation (SD) values of the cycle numbers are indicated as error bars. For the reaction mixture that did not show positive amplification signal in 55 thermal cycles, the *C*_q_ values were recorded as 55 to simplify the analysis. At an optimal Tm of 60 °C, the average *C*_q_ values of V600E MT-alleles in the tAS-PCR system using HQ-668, -666, and -663 were 26.25 ± 0.24, 30.79 ± 0.52, and 28.95 ± 0.50, respectively.

Using a specific amount of WT- and MT-gDNA (i.e., 50 ng) and the abovementioned ASPs targeting the V600E MT-alleles in the tAS-PCR system, the optimal Tm values was initially determined by using serial experiments under gradient Tm values ranging from 60 °C to 67 °C (Fig. 2). The results showed that the *C*_q_ values of both WT- and MT-alleles increased with higher Tm values. At a Tm >64 °C, both *C*_q_ values of the WT- and MT-alleles were significantly increased until no amplification occurred after 55 cycles. Moreover, at various Tm conditions (i.e., 60 °C–67 °C), the reaction system at 60 °C always showed lower *C*_q_ values and better S shapes of the fluorescent curves. Therefore, the Tm at 60 °C was determined as the optimal condition for all ASP oligonucleotides.

Next, the Δ*C*_q_ values between the WT- and MT-alleles were used to evaluate the priming specificity of various ASPs because more specific ASPs always have higher Δ*C*_q_ values. At the optimal Tm of 60 °C, HQ-663 showed a higher Δ*C*_q_ value (i.e., 3.24) compared to that of HQ-666 (i.e., 2.97) and -668 (i.e., 1.59). These findings indicate that the priming specificities could be enhanced by an artificially introduced mismatched nucleotide at the 3′-end of the ASP oligonucleotide. However, compared to the ASP that harbored a mismatched nucleotide at the *penultimate* positions (i.e., HQ-666), the ASP having a mismatched nucleotide at the *antepenultimate* positions (i.e., HQ-663) had better priming specificities because of its higher Δ*C*_q_ values.

**Figure 3:**
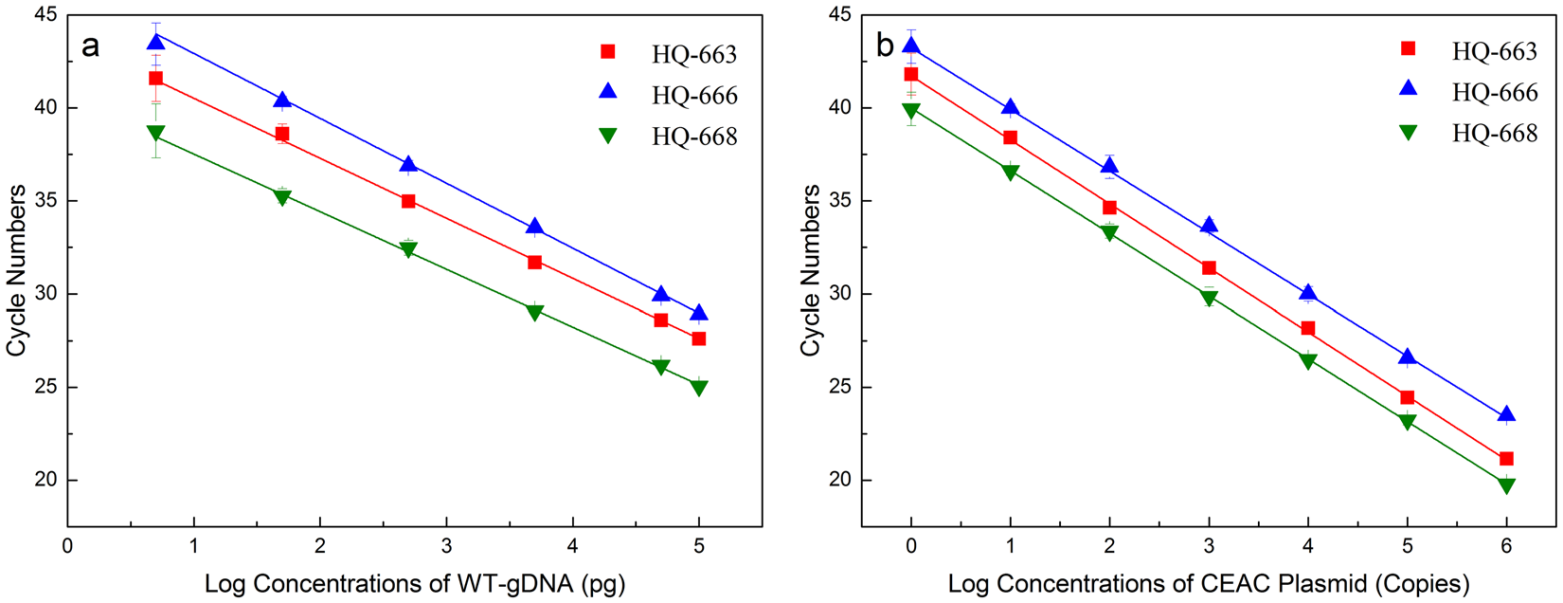
Sensitivity of tAS-PCR using various ASP oligonucleotides. For MT-gDNA (panel a), the amplification efficiencies of HQ-663, -666, and -668 were 101.87% (Y = −3.278X + 43.950; R^2^ = 0.9994), 96.77% (Y = −3.402X + 45.991; R^2^ = 0.9994), and 107.95% (Y = −3.145X + 40.840; R^2^ = 0.9992), respectively. For the CEAC plasmids (panel b), the amplification efficiencies of HQ-663, -666, and -668 were 95.20% (Y = −3.443X + 41.762; R^2^ = 0.9996), 99.83% (Y = −3.326X + 43.377; R^2^ = 0.9996), and 98.33% (Y = −3.363X + 39.988; R^2^ = 0.9999), respectively.

Finally, using serial concentrations of MT-gDNA (5 pg to 100 ng), both amplification efficiencies and analysis sensitivities were further evaluated for all ASP oligonucleotides. The results showed that all three ASPs had good amplification efficiencies, ranging from 96.77% to 107.95%, and were capable of detecting single-copy MT-alleles (Fig. 3a). Regardless of the concentration of the input MT-gDNA, the fully matched ASP (i.e., HQ-668) always showed the lowest *C*_q_ value. This observation indicated that the presence of the artificially introduced mismatched nucleotide decreases amplification efficiency. However, the priming specificities were enhanced based upon Δ*C*_q_ values between the WT- and MT-alleles (Figs 2 and 3). Compared to HQ-666 and -668, because of its higher specificity, similar amplification efficiency, and detection sensitivity, HQ-663 was selected as the optimal ASP oligonucleotide and thus utilized in the subsequent experiments.

### Eliminating non-specific amplifications using CEAC plasmids

Although mismatched bases were artificially introduced at the 3′-terminal positions of the ASP, the tAS-PCR still had significant amounts of non-specific amplification because the WT-gDNA had *C*_q_ values < 35 at a Tm of 60 °C (Fig. 2). As previously reported^14,16^, under the thermodynamic driving force of DNA polymerase, it is easy to trigger non-specific amplifications in tAS-PCR when there are no matched genotypic templates present in the reaction mixtures (Fig. 1a). Moreover, weak destabilization effects of terminal mismatch could further promote non-specific amplification^15^. Therefore, a novel method named cAS-PCR was developed to eliminate the non-specific amplifications and satisfy the thermodynamic driving force of DNA polymerase. Moreover, more than 50 thermal cycles were performed to enhance the detection of low-abundance alleles.

The cAS-PCR system (Fig. 1b) used an external CEAC plasmid that shared the same priming sequences as that of the targeted V600E. Therefore, both CEAC and V600E could be simultaneously amplified in the cAS-PCR system using a unique primer pair (i.e., HQ-663 and -675) that targets the V600E mutation. Moreover, the porcine LST could be used to discriminate the amplicons of the CEAC plasmids from the targeted V600E^18^. Because both CEAC plasmids and human gDNA are present in the cAS-PCR system, the ASP targeting the V600E will match at least one type of the input DNA (i.e., CEAC) and trigger its specific amplification regardless of the V600E genotype (Fig. 1b). Such amplification would perfectly fulfill the thermodynamic driving force of thermophilic DNA polymerase and thus dramatically reduce or eliminate the non-specific amplification between the ASP oligonucleotide and V600E WT-alleles that was frequently occurs in the tAS-PCR system^14,16^.

**Figure 4:**
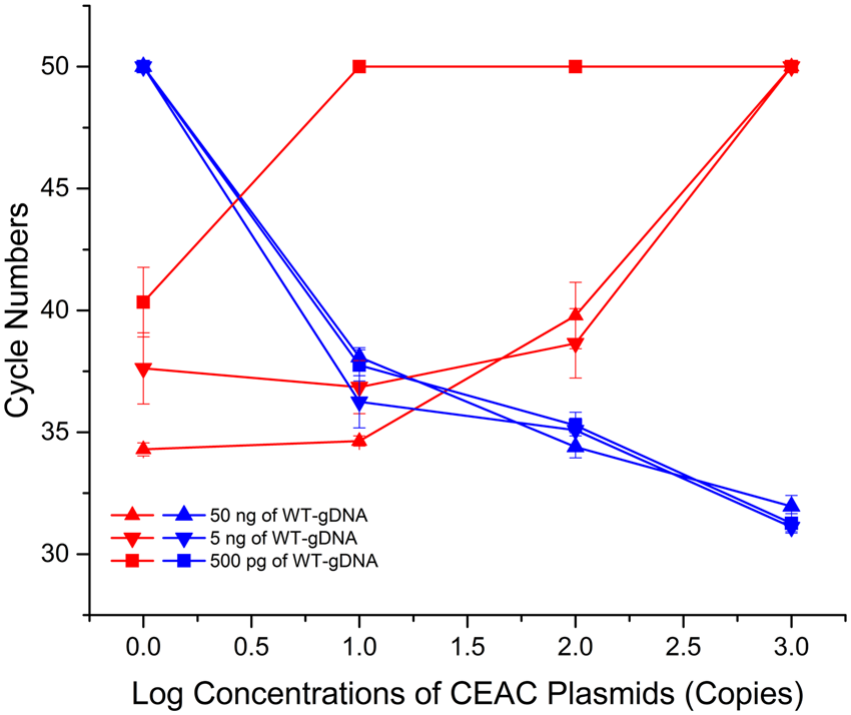
The suppressive effects of cAS-PCR for *BRAF* V600E WT-alleles. Experiments were performed with a reaction mixture containing the indicated concentrations of WT-gDNA and serial concentrations of CEAC plasmids. The *C*_q_ values of WT-gDNA and CEAC plasmids are indicated using red and blue lines, respectively. For the reaction mixture that did not show positive amplification signals after 50 thermal cycles, the *C*_q_ values were recorded as 50 to simplify the analysis. In the reaction without CEAC, the mean *C*_q_ values of WT-gDNA at final concentrations of 50 ng, 5 ng, and 500 pg were 34.30 ± 0.27, 37.62 ± 1.46, and 40.34 ± 1.43, respectively. In the cAS-PCR reactions containing 1,000 copies of the CEAC plasmid, the amplifications were eliminated after 50 cycles with any concentration of WT-gDNA.

To verify this hypothesis, various concentrations of WT-gDNA (ranging from 5 pg to 50 ng) was amplified in a reaction mixture containing serial copies of CEAC plasmid (0, 10, 100, and 1,000 copies; Fig. 4). Non-specific amplification of 5 pg or 50 pg WT-gDNA was completely suppressed (data not shown) under all conditions. However, with ≥500 pg WT-gDNA, non-specific amplification was suppressed in a CEAC dose-dependent (Fig. 4). The non-specific amplification of any tested concentration of WT-gDNA could only be completely suppressed when the CEAC copy number reached 1,000 (Fig. 4).

### Optimization of CEAC concentration in the cAS-PCR system

**Figure 5:**
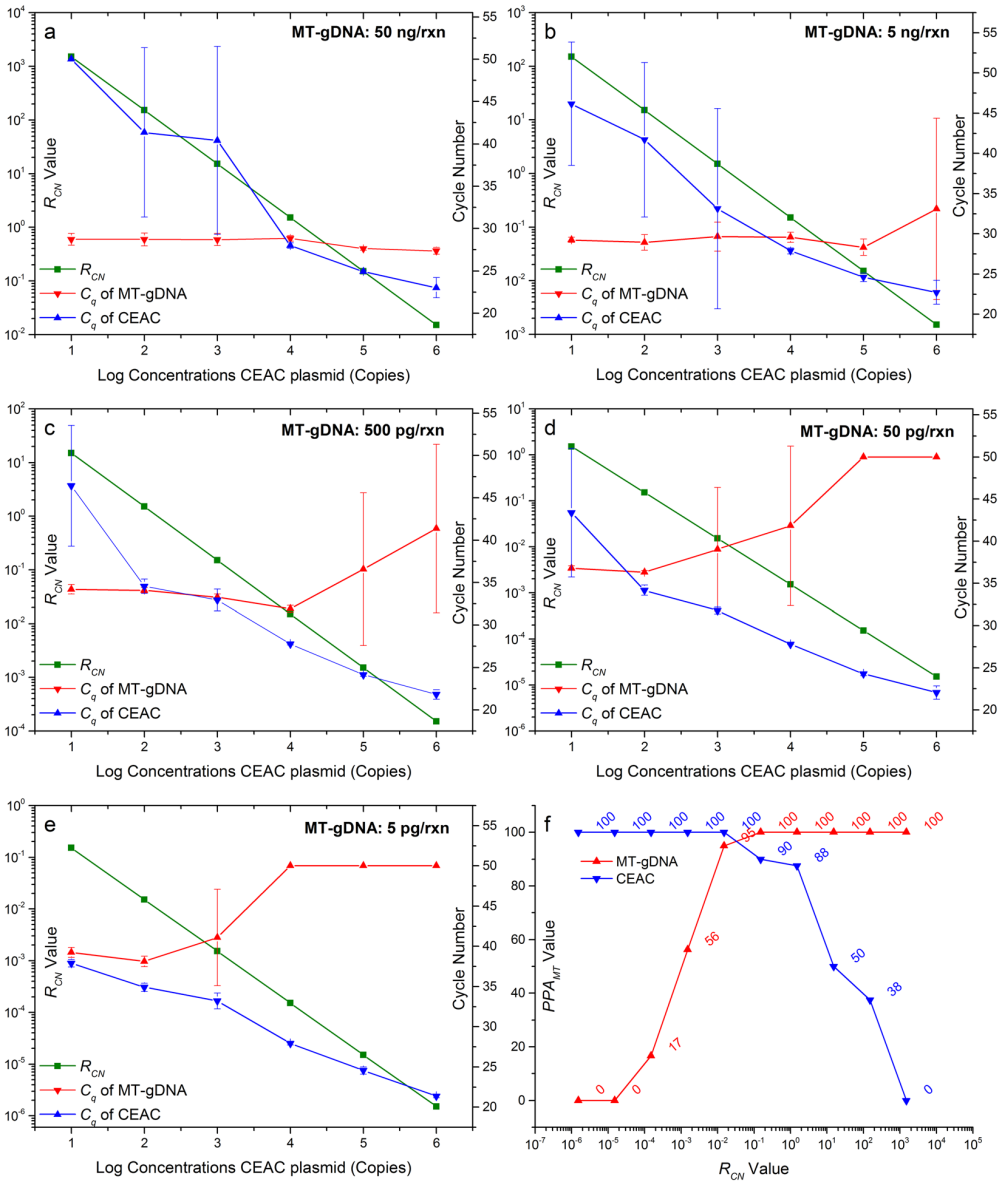
Optimizing the concentration of CEAC plasmids. The *C*_q_ values of MT-gDNA or CEAC plasmids were negatively correlated with their corresponding copy numbers. The *R*_CN_ was defined as the ratio of the copy number between MT-gDNA and CEAC (*R*_CN_ = *CN*_MT_/*CN*_CEAC_, in which *CN*_MT_ and *CN*_CEAC_ refer to the copy numbers of MT-gDNA and CEAC plasmids, respectively). The *PPA*_MT_ was defined as the percentage of samples with a positive amplification signal of MT-gDNA at certain *R*_CN_ or *P*_MT_ values, in which the *P*_MT_ indicated the copy number of MT-gDNA in the reaction mixture (*P*_MT_ = *CN*_MT_/[*CN*_MT_ + *CN*_CEAC_]). Panels a–e show the *R*_CN_ values (i.e., green lines), the *C*_q_ values of MT-gDNA (red lines) and CEAC plasmids (blue lines) at various amounts of MT-gDNA, ranging from 5 pg to 50 ng, indicating a 10-fold decrease, as indicated. Panel f shows the *PPA*_MT_ values at a specific *R*_CN_ for both MT-gDNA (red line) and CEAC plasmid (blue lines). Because the amount of human gDNA of each haplotype was ~3.3 pg, the copy numbers for the MT-gDNA used in panels a–e ranged from 1.52 × 10^4^ to 1.52 × 10^0^, indicating a 10-fold magnitude. Therefore, there are 10 *R*_CN_ values between serial copy numbers of the MT-gDNA (i.e., 1.52 × 10^4^ to 1.52 × 10^0^, indicating a 10-fold decrease) and CEAC plasmids (i.e., 1.00 × 10^1^ to 1.00 × 10^6^, indicating a 10-fold decrease), i.e., 1.52 × 10^−6^ to 1.52 × 10^3^, indicating a 10-fold decrease, as shown in the X-axis of panel f.

In cAS-PCR, although the preliminary results (Fig. 4) showed that the non-specific amplification of up to 50 ng WT-gDNA could be eliminated in the presence of 1,000 copies of the CEAC plasmid, it was unclear whether the amplification of the targeted V600E MT-alleles was also inhibited. Because the amplification of lower-abundance templates is always suppressed in the presence of a higher-abundance template in almost any PCR system, further optimization of CEAC concentration in the cAS-PCR was needed to avoid the suppressed amplification of targeted V600E MT-alleles. Therefore, serial concentrations of the CEAC plasmid (ranged from 1.0 × 10^1^ to 10^6^ copies) and MT-gDNA (5 pg to 50 ng; i.e., 1.52 × 10^0^ to 10^4^ copies) were co-amplified in cAS-PCR (Fig. 1b). Four parameters were used to evaluate the optimal concentration of CEAC plasmids: the *C*_q_ values of MT-gDNA or CEAC plasmids, the ratio of the copy number (*R*_CN_) between MT-gDNA and CEAC, the percentage of MT-gDNA (*P*_MT_) in the reaction mixture, and the percentage of samples with a positive amplification signal of MT-gDNA (*PPA*_MT_) at certain *R*_CN_ or *P*_MT_ values (Fig. 5).

Two batches of experiments, each conducted in duplicate, were performed to obtain a total of 1,200 datasets (Fig. 5). At the various conditions indicated in Fig. 5, the *C*_q_ values of the V600E MT-alleles and CEAC plasmids were positively and negatively correlated to the *R*_CN_ values, respectively. This result coincided with their corresponding total amount in the cAS-PCR. When the *P*_MT_ values were <0.01% (or *R*_CN_ < 1.5 × 10^−4^), no positive amplification of the V600E at any concentration of MT-gDNA was observed (Fig. 5f, Table S4). When the *P*_MT_ values were increased by 0.0151% (or *R*_CN_ = 1.5 × 10^−4^), the V600E MT-alleles were only amplified in 16.67% (2/12) of the samples (Fig. 5f, Table S1). However, when the *P*_MT_ values were increased by 1.4925% (or *R*_CN_ = 1.5 × 10^−2^), the *PPA*_MT_ values increased by 95% (19/20), even including 5 pg of MT-gDNA (Fig. 5f, Table S4). These results indicated that the amplification of targeted *BRAF* V600E MT-alleles might be suppressed by the high-abundance of CEAC plasmids. These findings were in agreement with other similar studies in which fewer concentrated sequences are often not amplified because the PCR favors the amplification of the predominant DNA type^24^. Therefore, there was need to optimize the concentration of CEAC plasmids based on following requirements: (a) non-specific amplification of WT-gDNA could be eliminated; and (b) effective amplification of low-abundance MT-gDNA could not be suppressed.

Further data analysis showed that under various conditions, MT-gDNA concentrations as low as 5 pg (i.e., a single-copy of the V600E MT-allele) could only be consistently amplified when ≤1,000 copies of CEAC were used (Fig. 5a–e). Moreover, the previous results indicated that the amplification of higher concentrations of WT-gDNA (i.e., 5 and 50 ng) could not be efficiently suppressed or eliminated when ≤100 copies of CEAC were present (Fig. 4). These results indicated that the optimal concentration of CEAC in the cAS-PCR was 1,000 copies. Such conditions could satisfy the requirements for optimal CEAC concentrations: (a) the non-specific amplification of WT-gDNA could be effectively eliminated at concentrations of 50 ng (Fig. 4) or higher (i.e., 100 ng; data not shown); (b) the amplification of MT-gDNA even as a single-copy could not be suppressed. Therefore, the optimal concentrations of CEAC plasmids in the cAS-PCR was determined as 1,000 copies and used in the subsequent experiments.

### Methodology parameters and the clinical application of cAS-PCR

**Figure 6:**
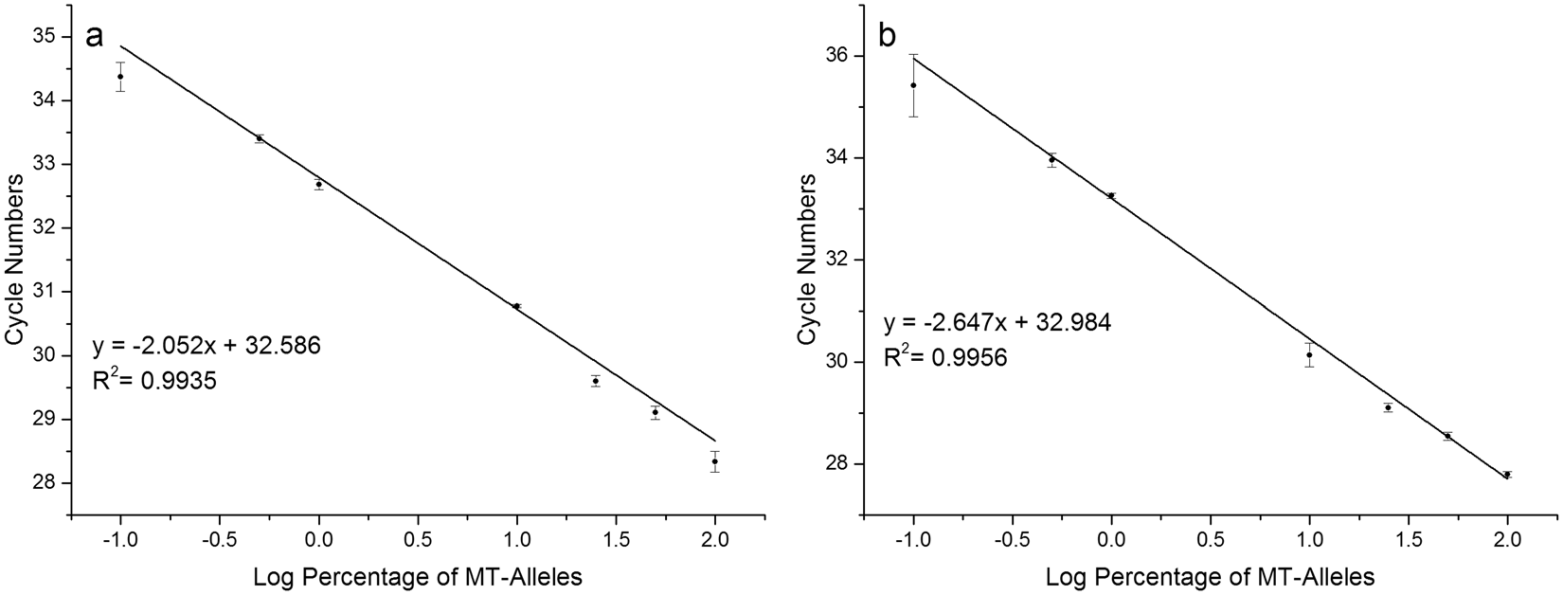
Standard curves showing the selectivity of cAS-PCR and rcAS-PCR. Panels a and b show the standard curves generated by plotting the average *C*_q_ values of MT-alleles from real-time cAS-PCR and rcAS-PCR, respectively, against the log percentages of V600E MT-alleles (0.1%, 0.5%, 1%, 10%, 25%, 50%, and 100%) present in 100 ng of WT- and MT-gDNA. The mean *C*_q_ values were obtained from Figures S1, respectively. These were determined automatically by using CFX Manager™ Software v3.1. The mean *C*_q_ values of CEAC plasmids were 31.21 ± 0.41 and 31.11 ± 2.62 in panels a and b, respectively; the mean C_q_ value of RIPC (i.e., *leptin*) was 27.58 ± 0.38 (panel b).

Using serial concentrations of MT-gDNA and under optimal reaction conditions (i.e., Tm at 60 °C, using ASP oligonucleotides of HQ-663, and 1,000 copies of CEAC plasmids), the results showed that the cAS-PCR system had the sensitivity to detect a single-copy of the V600E MT-allele (Figs 5e and 6a). To determine its selectivity, a concentration gradient of the MT-allele was prepared by mixing increasing concentrations of MT-gDNA with WT-gDNA. Our results showed that cAS-PCR always achieved consistent *C*_q_ values for the CEAC plasmids at 30.95 ± 0.61 under various concentrations of the V600E MT-alleles (Fig. S1). Moreover, the *C*_q_ values of the MT-alleles progressively and proportionally increased with the quantity of the V600E MT-alleles, indicating that the amplification of V600E WT-alleles was efficiently inhibited or eliminated in accordance to the principles of cAS-PCR. The cAS-PCR standard curve containing serial quantities of MT-alleles indicated that the current real-time cAS-PCR facilitates the detection of V600E MT-alleles with selectivity limitations as low as 0.1% (Figs 6b and S2).

During the preliminary analysis of the 50 FFPE samples, the V600E MT-alleles of only two samples were amplified. However, the mutant allele frequencies (4.0%; 2/50) were lower than that of previous reports^4–7^. Because the concentration of FFPE gDNA was not precisely determined, we speculate that abovementioned lower mutant frequencies might be attributable to the insufficiency in the amount of template used in the reaction. Therefore, the concentration of each FFPE DNA solution was determined using the *leptin* qPCR system (Fig. S2). Approximately 60% of the samples had concentrations of <10 ng/μL. Based on the results of the *leptin* qPCR, the amount of input gDNA was increased to 50–100 ng and further analyzed using both of current developed cAS-PCR system and commercial available reagent, i.e., the *BRAF* V600E Mutation Detection Kit (Amoy Diagnostics). Including the abovementioned two samples, a total of five samples were simultaneously determined as V600E mutations by using both methods. However, there was an additional sample that was determined as mutation-positive only by cAS-PCR, which indicated that the MT-allele percentage of this sample might be <1% because the lowest detection limit was 1% for that commercial available kit. Moreover, compared to the preliminary analysis without precisely controlling the total amount of input DNA, the results showed that the V600E MT-alleles were amplified from an additional four samples (i.e., 12.0%; 6/50). This indicated that the false-negative results might have also been generated by the cAS-PCR system, which was possibly due to the use of an insufficient amount of input gDNA. Although concentration determinations of the initial DNA solutions could prevent the generation of false-negative results such as using current *leptin* qPCR system, Quant-iT PicoGreen dsDNA Assay Kit (Invitrogen), or spectrophotometers, it was time-consuming and costly. Therefore, a novel method of rcAS-PCR was developed to eliminate false-negative results derived from the use of an insufficient amount of input DNA.

### Optimizing the final concentrations of oligonucleotides in the rcAS-PCR

In rcAS-PCR (i.e., cAS-PCR with RIPC; Fig. 1c), the amplification of RIPC could serve as a marker in monitoring the quantity of the input gDNA and therefore avoid false-negative results caused by the use of insufficient amounts of input gDNA. To avoid suppressing or eliminating the amplification of target V600E MT-alleles by RIPC, the Taguchi method was performed to optimize the final concentrations of each primer pair and TaqMan probe used for the rcAS-PCR reaction (Table S1).

According to the principles of the Taguchi method^19,20^, a modified L_16_(4^5^) orthogonal array was designed with 16 experiments using five factors each with four levels (Table S2). All calculations were performed as described in the Supplementary information, following the formulae described in our previous publication^14^. Only the *C*_q_ values of both MT-alleles and RIPC were considered because the amplification of the CEAC was consistent in all reactions (data not shown). The Δ*C*_q_ between the MT-alleles and RIPC was selected as the response variable (Table S3). In addition, the “smaller-the-better” equation was employed in calculating for the S/N ratio (*η*) using experimentally determined Δ*C*_q_ values^14,19,20^. The mean *η* for each level of each factor was used to determine the optimal level (i.e., the highest *S*/*N* ratio) and calculate the percent contribution (*P*_C_) of each factor (Table 2). The results showed that both primers and probes targeting the RIPC had the highest *P*_C_ values and significantly contributed to the lower Δ*C*_q_ values. Moreover, the probes targeting the MT-alleles also significantly affected the Δ*C*_q_ values. Based on the principle of the Taguchi method, the optimal levels of each factor are presented in the “Optimum” column of Table 2. Further tests confirmed these optimal conditions (Table 3), which were then used in subsequent experiments, which are described as follows: 0.5 μM for primer pair targeting both of V600E and CEAC; 0.5 μM for primer pair targeting RIPC; and 0.1, 0.1 and 0.25 μM for probes targeting V600E, CEAC and RIPC, respectively.

**Table 2:** The average signal-to-noise (*η*) of each level of each factor.

Factor	Level				Optimum	p	*P*_C_ (%)
	1	2	3	4			
A	−6.16	−5.58	−4.39	−**4**.**35**	0.50 μM	0.056	9.94%
B	−7.18	−5.03	−4.23	−**4**.**04**	0.50 μM	0.015	27.68%
C	−**3**.**59**	−4.99	−5.61	−6.29	0.10 μM	0.029	17.19%
D	−**4**.**78**	−4.94	−5.33	−5.43	0.10 μM	Pooled	Pooled
E	−7.24	−5.66	−4.14	−**3**.**44**	0.25 μM	0.010	38.56%
Error							6.63%

Factors A to B refer to primer pairs targeting V600E MT-alleles (HQ-663 and -675) and RIPC (HQ-329 and 330), respectively. Factors C to E refer to LST oligonucleotides targeting V600E MT-alleles (HQ-467), CEAC (HQ-648), and RIPC (HQ-1294), respectively.

**Table 3:** Confirmatory test conditions and the corresponding *S/N* ratios and mean responses, predicted *S/N* ratios and mean responses, and predicted 95% confidence intervals (CI).

Description	Results
*η* Observed	1.54
*η* Predicted	−0.05
Prediction error	1.59
*η* Confidence interval	±1.77
*η* Within CI (95%)	Yes^#^
Mean response observed	0.84
Mean response predicted	0.87
Mean response prediction error	−0.04
Mean response confidence interval	±0.22
Mean response within CI (95%)	Yes

^#^“Yes” indicates that the observed *S*/*N* ratio (*η*) or mean response (Δ*C*_qMR_) was within the corresponding prediction interval.

### Methodology parameters and the clinical application of the rcAS-PCR

As to the cAS-PCR system, serial concentrations of MT-gDNA with and without WT-gDNA were used to determine the sensitivity and selectivity of rcAS-PCR. The results showed that rcAS-PCR had a similar sensitivity (i.e., up to a single-copy of MT-gDNA) and selectivity (0.1%) to cAS-PCR (Figs 6 and S3). For the 50 FFPE samples, the rcAS-PCR achieved the same results as the cAS-PCR.

## Discussion

AS-PCR is one of the most commonly used methods in detecting single-base variations, including oncogene mutations (e.g., *BRAF* V600E), that can be used to predict the responses of individualized treatments^11–13^. Some commercially available AS-PCR kits targeting *BRAF* V600E are currently used in the clinic such as the *BRAF* RGQ PCR Kit (Qiagen) and the *BRAF* V600E Mutation Detection Kit (Amoy Diagnostics). Because of intra-tumor heterogeneity, the number of mutated cancer cells in clinically available tissue samples is often relatively low, and thus the gDNA extractions usually generate predominantly WT-gDNA. In recent years, various studies have indicated that higher-selectivity mutation analyses are more useful in identifying patients with poor responses to anti-EGFR antibody therapy in mCRC^25–31^. Therefore, the development of a reliable and selective method for detecting low-abundance *BRAF* mutations could be useful in clinical screening to identify mCRC patients who are predicted to elicit a poor response to anti-EGFR antibody therapies. However, AS-PCR is strongly associated with two technical issues, namely, the non-specific amplification of ASP oligonucleotides against non-matched alleles (e.g., Figs 1 and 2) and the relative lower selectivity for the detection of low-abundance MT-alleles (e.g., only ~1–5% of *BRAF* V600E MT-alleles could be detected using commercial kits and other published assays; and the false-positive signal of WT-alleles could be presented at about 40 amplification cycles)^11–13^. Although other strategies in detecting *BRAF* V600E at much higher specificity (up to 100%) and selectivity (≥0.01%) such as WT-blocking PCR, BEAMing, or digital PCR are currently available, its relatively higher cost due to the use of expensive oligonucleotides (e.g., PNA, or LNA), droplet chips, or instruments has limited their clinical applications^32–34^.

Compared with previous publications and commercial available kits targeting *BRAF* V600E with AS-PCR methods^11–13^, three strategies were used to suppress or eliminate the non-specific amplification of ASP oligonucleotides against non-matched alleles in the present study. Then, various AS-PCRs, including cAS-PCR and rcAS-PCR, were developed to satisfy the clinical requirements in terms of specificity and selectivity. The three strategies were (Fig. 1): (a) modifying AS-PCR (i.e., an artificial mismatched nucleotide was introduced at the 3′-end of the ASP oligonucleotides (Table 1), (b) introducing CEAC into the reaction mixture (i.e., cAS-PCR) to satisfy the thermodynamic driving force of the thermophilic DNA polymerase, and (c) introducing RIPC into the reaction mixture (rcAS-PCR) to monitor the amount of input sample DNA.

Firstly, an artificial mismatched nucleotide was introduced at the *penultimate* (HQ-666) or *antepenultimate* (HQ-663) nucleotide of the ASP oligonucleotides targeting *BRAV* V600E to enhance the specificity of the tAS-PCR. Under various conditions (e.g., gradient Tm and serial concentrations of WT- or MT-gDNA), HQ-663 always achieved better *C*_q_ values and a larger Δ*C*_q_ between WT- and MT-gDNA than HQ-666 (Figs 2 and 3). These results indicated that HQ-663 had a better amplification efficiency and specificity than HQ-666. To the best of our knowledge, this is the first systematically comparative study on modified ASP oligonucleotides with an artificially introduced mismatched nucleotide in the *penultimate* or *antepenultimate* position. Although further experiments are needed to confirm these results, the current study suggests that introducing the mismatched nucleotide in the *penultimate* position is a better strategy than using the *antepenultimate* position. Therefore, HQ-663 was selected as the ASP oligonucleotides to target the *BRAF* V600E MT-alleles, which in turn was used to develop the current cAS- and rcAS-PCR systems.

Although the ASP oligonucleotides used in the tAS-PCR systems had an artificial mismatched nucleotide, the results still indicated that non-specific amplification between the ASP oligonucleotides and V600E WT-alleles could not be eliminated. This might be associated with the thermodynamic driving force of the thermophilic DNA polymerases^14,16^. In the tAS-PCR system, when no V600E MT-alleles were present in the reaction mixture (i.e., WT-gDNA), non-specific amplification between the ASP oligonucleotides and non-matched alleles (i.e., WT-alleles) was easily triggered because the PCR system was producing amplicons based on the thermodynamic driving force of the DNA polymerase^14,16^. However, in the cAS-PCR system, an external cross-species fragment (i.e., CEAC) sharing the same priming sequences as the targeted V600E MT-alleles was artificially introduced into the reaction mixture. Under such conditions, a specific amplification reaction will always occur between the ASP oligonucleotides and CEAC regardless of the V600E genotype, thus satisfy the thermodynamic driving force and then reducing or eliminating non-specific amplification that commonly occurs in a tAS-PCR system (Fig. 1a and b)^13^. Another benefit of the cAS-PCR system was that the false-negative results caused by residual PCR amplification inhibitors in extracted DNA solutions from FFPE sections were eliminated^35^.

When serial concentrations of the MT-gDNA and CEAC plasmids were co-amplified in the cAS-PCR system, the amplification of MT-gDNA was suppressed or eliminated by significantly higher concentrations of the external CEAC fragments (Fig. 5), which was consistent with the principle of traditional competitive PCR^36,37^. However, when the concentration of the CEAC plasmid was too low (e.g., 100 copies), the non-specific amplification between the ASP oligonucleotides and WT-gDNA could not be eliminated (Fig. 4), which in turn might generate false-positive results. To the best of our knowledge, this is the first report that presents a systematically comparative study on the relationships between targeted templates and external cross-species fragments that could satisfy the thermodynamic driving force of thermophilic DNA polymerase^14,16^. Our findings indicate that there is a need to optimize the concentration of external fragments to avoid suppressing the amplification of the target templates.

Based on the optimization experiments (Figs 4 and 5), 1,000 copies of CEAC plasmid was determined as the optimal condition for cAS-PCR. Under such conditions, the mean *C*_q_ values of CEAC plasmids were kept consistent at various amounts of inputted MT-gDNA (Figs 5 and S1). According to the principle of traditional competitive PCR^36,37^, the *C*_q_ values of MT-gDNA or CEAC plasmids at certain concentrations could be changed according to their relative amount ratios (i.e., *R*_*CN*_ in the present study) because both templates would compete for the primers in the current cAS-PCR system (Fig. 1). However, the results showed that when the CEAC plasmids were lower than 1000 copies (i.e., 10^1^ or 10^2^ copies in Fig. 5), the *C*_q_ values of MT-gDNA at certain concentrations were kept consistent at various amounts of inputted CEAC plasmids, whereas the *C*_q_ values of CEAC plasmids at certain concentrations were increased at dose depended on the amounts of inputted MT-gDNA. Moreover, when the CEAC plasmids were higher than 1000 copies (i.e., 10^4^, 10^5^ or 10^6^ copies in Fig. 5), the *C*_q_ values of CEAC plasmids at certain concentrations were kept consistent at various amounts of inputted MT-gDNA, whereas the *C*_q_ values of MT-gDNA at certain concentrations were increased at dose depended on the amount of inputted CEAC plasmids. For example, as low as 5 pg of MT-gDNA could only be detected when the CEAC plasmids were equal or lower than 1000 copies (Fig. 5e). These results indicated that there were balance points between target and competitor in real-time competitive PCR (e.g., the current cAS-PCR) in which the *C*_q_ values of competitor (e.g., CEAC plasmids in the present study) could keep consistent at various amount of inputted targeted templates (e.g., MT-gDNA in the present study).

Under aforementioned optimized conditions, the cAS-PCR had the sensitivity of a single-copy (5 pg of gDNA) and a selectivity of 0.1% to detect V600E MT-alleles. Compared to other published and clinical available AS-PCR systems^11–13^, the observed relative higher selectivity (i.e., 0.1%) of the current cAS-PCR might be associated with its higher thermal cycle numbers (i.e., 50 cycles). Because non-specific amplification of the ASP oligonucleotides could not be efficiently reduced or eliminated, previous studies have employed only a limited number of thermal cycles (i.e., 35 to 40 cycles) in the AS-PCR system^11,12^. Using this particular setting, the low-abundance templates (e.g., <0.1%) were not efficiently amplified because higher-abundance templates were more readily bound and primed by the primers. However, with the extended thermal cycles (e.g., 50 cycles in the current cAS-PCR system), there was a higher probability for binding and priming between primers and low-abundance templates. This might also explain why the single-copy amplification of the targeted V600E MT-alleles could not be suppressed or eliminated by competitive CEAC plasmids at concentrations of 1,000-fold higher, and then to get higher selectivity (i.e., 0.1%) under excess WT-alleles.

Despite the observed sensitivity and selectivity of the cAS-PCR system, the generation of false-negative due to the use of an insufficient amount of input gDNA could not be circumvented. Therefore, in the present study, a third strategy was integrated in the rcAS-PCR, in which RIPC fragments (i.e., *leptin*) were co-amplified with the V600E MT-alleles and CEAC plasmids (Fig. 1c). Moreover, the Taguchi method was used to optimize the concentrations of various oligonucleotides used in the rcAS-PCR to avoid suppressing the amplification of the targeted V600E MT-alleles by the RIPC fragments^14,19,20^.

Regardless of the PCR methodology, optimization demands an investigation of the interaction between multiple variables and involves unusually large experiments^16,38^. Traditionally, complete optimization can be achieved by testing each variable independently by the factorial method, which is costly and time-consuming^16,38^. Therefore, in the current study, the Taguchi method, a Design of Experiment was used to optimize the conditions and evaluate the characteristics of rcAS-PCR^14,19,20,38^. The Taguchi method was selected because it provides a systematic approach in designing experiments, and consequently, has been the most widely used technique in automotive and electronics industrial design for the last two decades^19,20,39–44^. One of the important steps in Taguchi’s technique is the selection of orthogonal arrays, which helps in determining the optimal level for each parameter and establishes the relative importance of individual parameters using a small set of all possibilities in a minimal number of experiments^19,20,39^. Following the principle of the Taguchi method, a modified L_16_(4^5^) orthogonal array was selected to optimize the conditions and evaluate the characteristics of rcAS-PCR. Compared to factorial methods that require 1,024 (4^5^) experiments, only 16 experiments were performed to optimize the rcAS-PCR reaction parameters, which dramatically increased the efficiency of the optimization and evaluation (Tables S1 and S2). The results showed that primers and probes targeting RIPC had the highest *P*_C_ values, with *p* values < 0.05 (Table 2). This indicates that the amplification of RIPC had a significant effect upon the amplification efficiency of the targeted V600E. Consideration of the amplification relationships between the CEAC and V600E or RIPC and V600E (Figs 4 and 5; Table 2) indicate that it is necessary to optimize the amplification of any external or internal reference fragment to avoid suppressing the amplification of target genes.

Based on the optimization using Taguchi method, the rcAS-PCR exhibited a similar sensitivity and selectivity as cAS-PCR. However, rcAS-PCR had additional advantages over cAS-PCR such as circumventing the false-negative results caused by an insufficient amount of input templates.

## Conclusion

Although artificially mismatched nucleotides could be introduced into ASP oligonucleotides to enhance their specificity in the tAS-PCR system, the non-specific amplification of opposite genotypic alleles could not be completely suppressed or eliminated, which in turn reduces both the sensitivity and selectivity of the tAS-PCR system. The introduction of CEAC plasmids into the tAS-PCR system to satisfy the thermodynamic driving force of thermophilic DNA polymerase prevented the occurrence of non-specific amplifications in the cAS-PCR system. Moreover, cAS-PCR had the additional advantage of eliminating the false-negative results caused by various PCR inhibitors that might be present in the clinical DNA samples. The rcAS-PCR system was developed further to circumvent the false-negative results caused by using an insufficient amount of input template, which often occurs during cAS-PCR. Based on the optimized reaction parameters determined using serial experiments, both cAS-PCR and rcAS-PCR had the sensitivity to detect a single-copy of the human *BRAF* V600E MT-allele, with a selectivity of 0.1%. Therefore, these methods might serve as powerful and robust routine genotyping tools with clinical applications. In our laboratory, similar rcAS-PCR assays targeting other oncogenic mutations that play important roles in personalized treatments such as *KRAS* and *EGFR* genes are being developed.

## Electronic supplementary material

Supplementary Methods, Tables and Figures
