# Supplementary material for: Improved detection of BRAF V600E using allele-specific PCR coupled with external and internal controllers
Source: Sci Rep. 2017 Oct 23;7:13817. doi: 10.1038/s41598-017-14140-2 (PMC5653796; doi:10.1038/s41598-017-14140-2)
Supplement: Supplementary file 1 — Supplementary Methods, Tables and Figures [file 41598_2017_14140_MOESM1_ESM.pdf]

Improved detection of *BRAF* V600E using allele-specific PCR coupled with external and internal controllers

Zhao Yang[1#], Na Zhao[1#], Dong Chen[2], Kun Wei[1], Ning Su[1], Jun-Fu Huang[1], Han-Qing Xu[1], Guang-Jie Duan[3], Wei-Ling Fu[1]*, Qing Huang[1]*

[1]Department of Laboratory Medicine, Southwest Hospital, Third Military Medical University, Chongqing, 400038, P. R. China.

[2]Department of Laboratory Medicine; 302 hospital of PLA, Chongqing, 100039, P. R. China.

[3]Institute of Pathology and Southwest Cancer Center, Southwest Hospital, Third Military Medical University, Chongqing, 400038, P. R. China.

Running title: cAS-PCR and rcAS-PCR targeting *BRAF*

[#] Equal Contributions

[*]Corresponding Author:

Prof. Qing Huang, PhD, MD

Tel: +86 23 6546 0802; Fax: +86 23 6546 0909; Email: Dr.Q.Huang@gmail.com

Prof. Wei-Ling Fu, PhD, MD

Tel: +86 23 6546 0802; Fax: +86 23 6546 0909; Email: fwl@tmmu.edu.cn

The Taguchi method

The experiment design, data calculating and analysis method were performed following our previous publications [1]. According to the principles of Taguchi method [2,3], the ΔC_q values between MT-alleles and RIPC amplicons was selected as the response variable. Five factors were optimized at four levels (Table S2). Therefore, an $L_{16}(4^5)$ orthogonal array was designed (Table S2, S3). All calculations of the Taguchi method were done by inputting the formulae described below (Eq. 1-7) and corresponding values into a Microsoft® Excel spreadsheet.

An optimized assay should have the lowest possible ΔC_q values; therefore, the "smaller-the-better" equation (Eq. 1) was employed to calculate the S/N ratio (η) using an experimentally determined ΔC_q values. The "smaller-the-better" *S/N* ratio were calculated as follows [1-3]:

$$\eta = -10\log_{10}\left[\frac{1}{r}\sum_{i=1}^{r} y^2\right] \quad \text{(Eq. 1)}$$

where η= the *S/N* ratio, r=the number of repeated runs, y=the response (i.e., ΔC_q).

The percent contribution (P_C) of each factor to the total variations observed in each experiment was calculated as follows (Eq. 2 to 4) [1-3]:

$$SS_T = \sum_{i=1}^{n}(\eta_i - \bar{\eta})^2 \quad \text{(Eq. 2)}$$

$$SS_X = \sum_{i=1}^{f} R_{Xi}\times(\bar{\eta}_{Xi} - \bar{\eta})^2 \quad \text{(Eq. 3)}$$

$$P = \frac{SS_X - V_E\times dof_X}{SS_T} \quad \text{(Eq. 4)}$$

where SS_T=the total sum of squares, n=the number of experiments in the orthogonal array, η_i=the *S/N* ratio of each experiment, and $\bar{\eta}$=the average *S/N* ratio of all experiments; SS_X=the sum of squares of the signal-to-noise ratio of factor X, in which f=the number of factors, R_{Xi} is the number of runs conducted at level i of factor X; $\bar{\eta}_{Xi}$=the average *S/N* ratio for each level of each factor; V_E=the variance of error; dof_X=the degree of freedom of factor X. The F-ratio and p-value of each factor were further analyzed by Analysis Of Variance (ANOVA).

To predict the *S/N* ratio of a reaction carried out with optimal conditions, the following equation was used (Eq. 5) [1-3]:

$$\eta_{opt} = \eta_m + \sum_{i=1}^{f}(\bar{\eta}_i - \eta_m) \quad \text{(Eq. 5)}$$

where η_m=the overall mean of *S/N* ratios at the optimal level for all factor combined, $\bar{\eta}_i$=the mean *S/N* ratio at optimal levels of each factor i.

The 95% confidence interval at optimal conditions was calculated as follows (Eq. 6) [1-3]:

$$\mathrm{CI} = \sqrt{\frac{F_{95\%,1,dof_E}\times V_E}{n_{eff}}} \quad \text{(Eq. 6)}$$

where $F_{(95\%,1,dof_E)}$=the first F value with the degree of freedom equal to 1 and the degree of freedom of error (dof_E) as the second degree of freedom at 95% confidence; V_E=the variance of error; n_{eff}= the effective sample size determined by $N/(1+dof_{TF})$, where N=the total number of experiments, and

dof_{TF}=the degree of freedom of all factors combined.

As confirmation, the *S*/*N* ratio should fall within the 95% confidence interval, given that a prediction model is suitable. The confidence interval was calculated using the following equation (Eq. 7) [1-3]:

$$\mathrm{CI} = \sqrt{F_{95\%,1,dof_E} \times V_E \left(\frac{1}{n_{eff}} + \frac{1}{n_{conf}}\right)} \quad \text{(Eq. 7)}$$

where n_{conf}=the number of confirmatory tests conducted.

References

1. Chen, D. et al. Enhanced specificity of TPMT*2 genotyping using unidirectional wild-type and mutant allele-specific scorpion primers in a single tube. PloS one 9, e91824, doi:10.1371/journal.pone.0091824 (2014).

2. Ross, P.J. Taguchi techniques for quality engineering: loss function, orthogonal experiments parameter and tolerance design. (Productivity Press Inc, 1986).

3. Taguchi, G. Introduction to quality engineering: designing quality into products and processes (trans: Organization AP). (Productivity Press Inc, 1986).

Table S1. Factors and their corresponding levels in the Taguchi method.

Factors	Oligonucleotides	Properties	Targeted	Level (μM)			
				**1**	**2**	**3**	**4**
**A**	HQ-663 & -675	Primer pair	*BRAF* & CEAC	0.20	0.30	0.40	0.50
**B**	HQ-329 & -330	Primer pair	RIPC	0.20	0.30	0.40	0.50
**C**	HQ-467	LST	*BRAF*	0.10	0.15	0.20	0.25
**D**	HQ-648	LST	CEAC	0.10	0.15	0.20	0.25
**E**	HQ-1294	LST	RIPC	0.10	0.15	0.20	0.25

The copy numbers of CEAC plasmid were kept consistent at 1,000 copies in each assay because that was the optimal level determined.

Table S2. The $L_{16}(4^5)$ orthogonal array used to optimize rcAS-PCR.

Experiment.	Levels				
	A	B	C	D	E
**1**	1	1	1	1	1
**2**	1	2	2	2	2
**3**	1	3	3	3	3
**4**	1	4	4	4	4
**5**	2	1	2	3	4
**6**	2	2	1	4	3
**7**	2	3	4	1	2
**8**	2	4	3	2	1
**9**	3	1	3	4	2
**10**	3	2	4	3	1
**11**	3	3	1	2	4
**12**	3	4	2	1	3
**13**	4	1	4	2	3
**14**	4	2	3	1	4
**15**	4	3	2	4	1
**16**	4	4	1	3	2

Table S3. Results of the $L_{16}(4^5)$ orthogonal array.

Exp.	η	Mean	SD	ΔC_q values between MT-alleles and RIPC					
				No. 1	No. 2	No. 3	No. 4	No. 5	No. 6
**1**	–8.48	2.65	0.16	2.77	2.91	2.50	2.59	2.58	2.55
**2**	–6.31	2.07	0.11	2.20	1.88	2.04	2.05	2.06	2.16
**3**	–4.99	1.76	0.25	1.35	1.77	1.86	1.96	1.61	2.02
**4**	–4.88	1.75	0.14	1.90	1.80	1.80	1.76	1.48	1.75
**5**	–6.04	2.00	0.08	1.96	1.88	1.99	2.05	2.03	2.12
**6**	–3.28	1.45	0.16	1.18	1.47	1.58	1.47	1.38	1.63
**7**	–6.06	2.01	0.14	1.75	2.14	1.98	2.07	2.03	2.06
**8**	–6.92	2.22	0.13	1.95	2.33	2.21	2.28	2.24	2.28
**9**	–7.79	2.45	0.11	2.66	2.43	2.34	2.43	2.41	2.43
**10**	–7.81	2.45	0.26	2.40	1.95	2.60	2.61	2.51	2.60
**11**	–0.11	1.01	0.09	0.97	0.98	1.10	0.89	1.13	1.00
**12**	–1.87	1.24	0.10	1.10	1.25	1.39	1.27	1.17	1.24
**13**	–6.43	2.09	0.16	2.21	1.89	2.21	2.01	2.28	1.95
**14**	–2.72	1.36	0.16	1.41	1.16	1.59	1.46	1.28	1.26
**15**	–5.76	1.94	0.12	2.04	2.09	1.90	1.96	1.86	1.78
**16**	–2.48	1.33	0.08	1.27	1.22	1.45	1.38	1.33	1.31

Table S4. Relationship between the copy number ratio (R_{CN}), percentage of MT-gDNA (P_{MT}), and positive amplification of MT-gDNA and CEAC plasmid.

		MT-gDNA				CEAC			
R_{CN}	P_{MT} (%)	Pos	Neg	Total	P (%)	Pos	Neg	Total	P (%)
1.52×10^{1}	99.9340	4	0	4	100.00	0	4	4	0.00
1.52×10^{2}	99.3443	8	0	8	100.00	3	5	8	37.50
1.52×10^{1}	93.8086	12	0	12	100.00	6	6	12	50.00
1.52×10^{0}	60.2410	16	0	16	100.00	14	2	16	87.50
1.52×10^{-1}	13.1579	20	0	20	100.00	18	2	20	90.00
1.52×10^{-2}	1.4925	19	1	20	95.00	16	0	16	100.00
1.52×10^{-3}	0.1513	9	7	16	56.25	16	0	16	100.00
1.52×10^{-4}	0.0151	2	10	12	16.67	12	0	12	100.00
1.52×10^{-5}	0.0015	0	8	8	0.00	8	0	8	100.00
1.52×10^{-6}	0.0002	0	4	4	0.00	4	0	4	100.00

Figure S1. Selectivity of the cAS-PCR system targeting *BRAF* V600E MT-alleles.

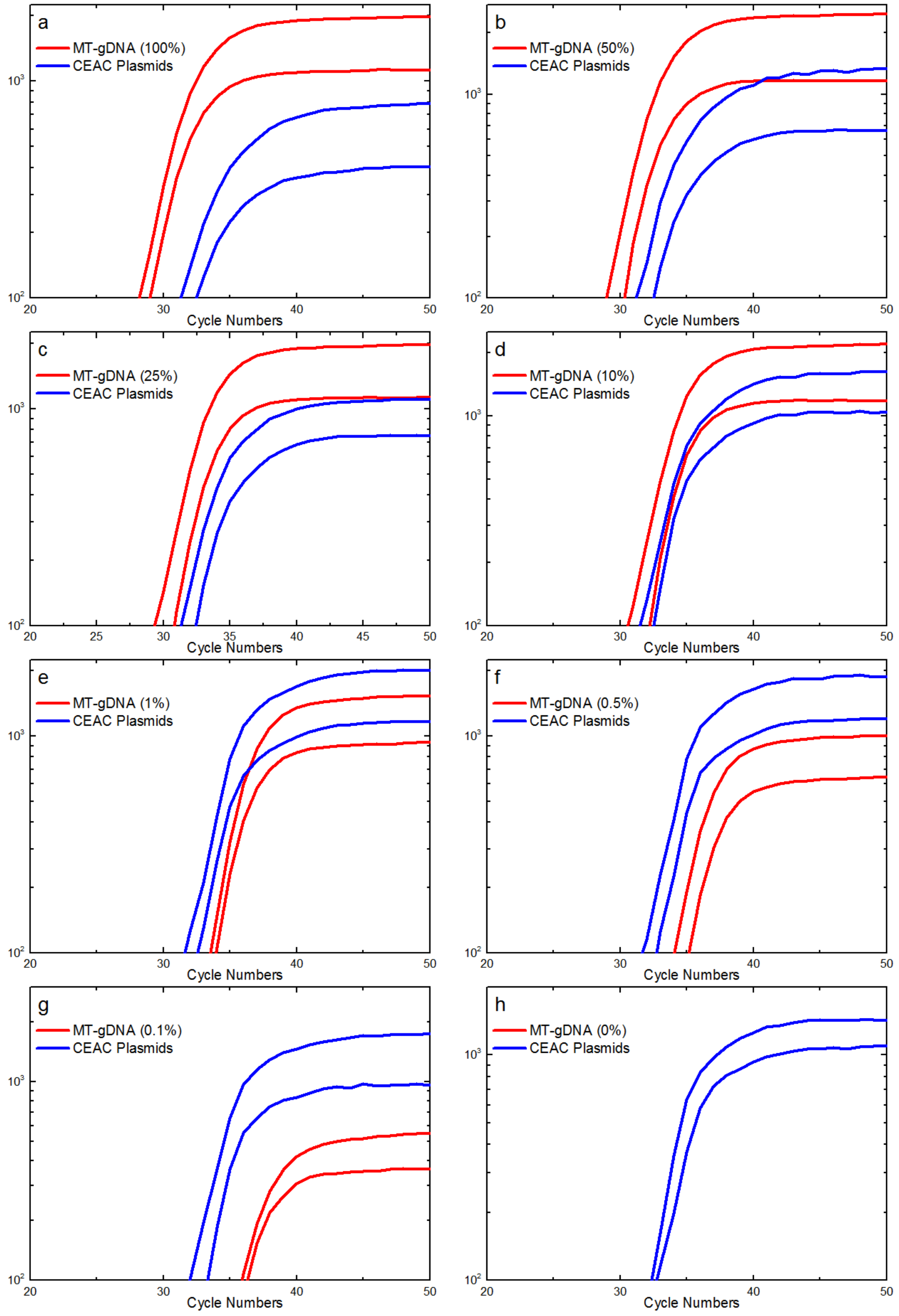

Panels a-h show the amplification curves of real-time cAS-PCR in which the blue and red lines indicate the fluorescent signal of MT-gDNA and CEAC plasmids, respectively. In a 20-μL reaction mixture, increasing quantities of MT-gDNA were spiked into samples containing WT-gDNA to give a total of 50 ng of gDNA. The gDNA was used to prepare templates containing increasing quantities of mutations (0.1%, 0.5%, 1%, 10%, 25%, 50%, and 100%) as indicated to evaluate the selectivity of the cAS-PCR system. The C_q values of the 1,000 copies of CEAC plasmid present in the cAS-PCR system remained consistent in various conditions.

Figure S2. Standard curves used to quantify gDNA from both cell lines and FFPE sections.

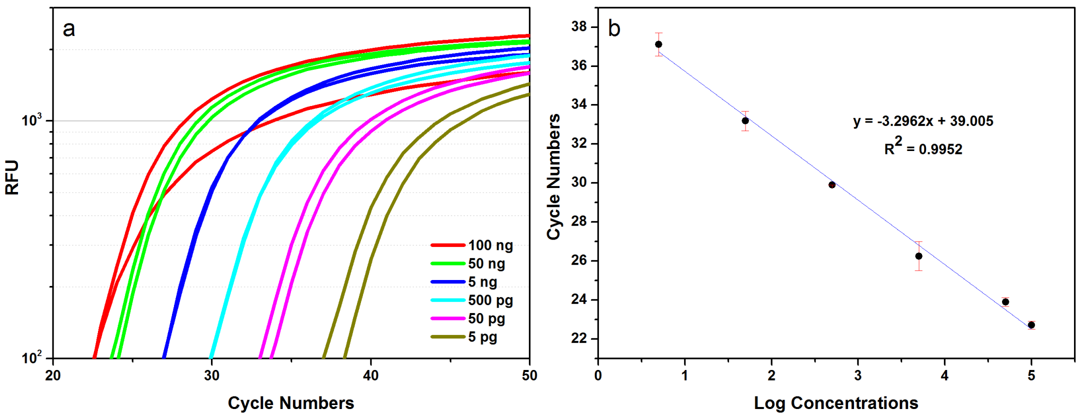

Panel a shows the amplification curves of *leptin* in a qPCR system using serial concentrations of standard human gDNA (Promega; 100 ng, 50 ng, 5 ng, 500 pg, 50 pg, and 5 pg) as the template. Panel b shows the standard curve generated from panel a, which was used to quantify the extracted DNA solutions.

Figure S3. Selectivity of the rcAS-PCR system targeting *BRAF* V600E MT-alleles.

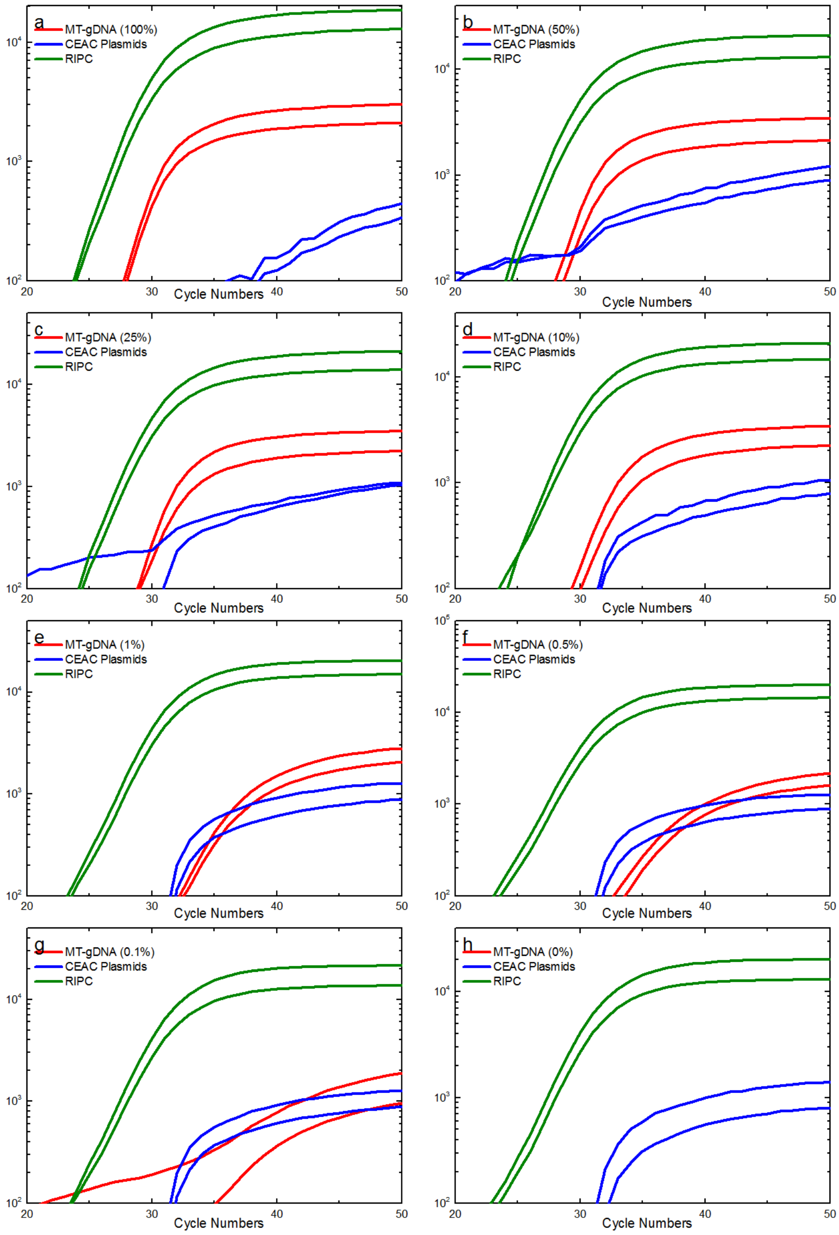

Panels a - h show the amplification curves of real-time rcAS-PCR in which the blue, red, and green lines indicate the fluorescent signals of MT-gDNA, CEAC plasmid, and RIPC, respectively. In a 20-μL reaction mixture, increasing quantities of MT-gDNA were spiked into samples containing WT-gDNA to give a total of 50 ng of gDNA. The resulting gDNA was used to prepare templates containing increasing quantities of mutation (0.1%, 0.5%, 1%, 10%, 25%, 50%, and 100%), as indicated, to evaluate the selectivity of the cAS-PCR system. The C_q values of the CEAC plasmids and RIPC presented in the rcAS-PCR system were kept consistent under the various conditions.
